# 2-{[4-(Pyridin-2-yl)pyrimidin-2-yl]sulfan­yl}acetic acid

**DOI:** 10.1107/S1600536811039791

**Published:** 2011-10-12

**Authors:** Lin Wang, Hua-Ze Dong

**Affiliations:** a Deparment of Chemistry and Chemical Engineering, Hefei Normal University, Hefei 230061, People’s Republic of China

## Abstract

In the title mol­ecule, C_11_H_9_N_3_O_2_S, the pyridine and pyrimidine rings are almost parallel [dihedral angle = 6.7 (1)°]. In the crystal, adjacent mol­ecules are joined by O—H⋯N and C—H⋯O hydrogen bonds, leading to the formation of a sheet parallel to (10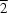).

## Related literature

For details of the synthesis and general background, see: Dong *et al.* (2009 ▶); Wang (2011 ▶). For the crystal structures of coord­ination complexes with related ligands, see: Du *et al.* (2004 ▶); Zhu *et al.* (2009 ▶).
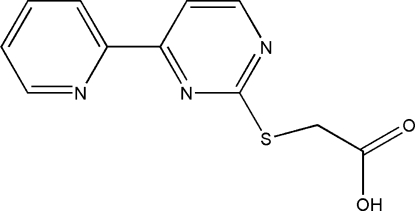


## Experimental

### 

#### Crystal data

C_11_H_9_N_3_O_2_S
*M* *_r_* = 247.28
Monoclinic, 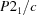

*a* = 6.5722 (2) Å
*b* = 22.4650 (8) Å
*c* = 7.4314 (2) Å
β = 93.237 (2)°
*V* = 1095.45 (6) Å^3^
*Z* = 4
Mo *K*α radiation
μ = 0.29 mm^−1^
*T* = 291 K
0.28 × 0.20 × 0.18 mm

#### Data collection

Bruker SMART CCD area-detector diffractometer
Absorption correction: multi-scan (*SADABS*; Bruker, 2000 ▶) *T*_min_ = 0.920, *T*_max_ = 0.950
10868 measured reflections
2524 independent reflections
2116 reflections with *I* > 2σ(*I*)
*R*_int_ = 0.022

#### Refinement

*R*[*F* ^2^ > 2σ(*F* ^2^)] = 0.035
*wR*(*F* ^2^) = 0.096
*S* = 1.05
2524 reflections
155 parameters
H-atom parameters constrained
Δρ_max_ = 0.21 e Å^−3^
Δρ_min_ = −0.24 e Å^−3^

### 

Data collection: *SMART* (Bruker, 2000 ▶); cell refinement: *SAINT* (Bruker, 2000 ▶); data reduction: *SAINT*; program(s) used to solve structure: *SHELXTL* (Sheldrick, 2008 ▶); program(s) used to refine structure: *SHELXTL*; molecular graphics: *SHELXTL*; software used to prepare material for publication: *SHELXTL*.

## Supplementary Material

Crystal structure: contains datablock(s) I, global. DOI: 10.1107/S1600536811039791/kj2186sup1.cif

Structure factors: contains datablock(s) I. DOI: 10.1107/S1600536811039791/kj2186Isup2.hkl

Supplementary material file. DOI: 10.1107/S1600536811039791/kj2186Isup3.cml

Additional supplementary materials:  crystallographic information; 3D view; checkCIF report

## Figures and Tables

**Table 1:** Hydrogen-bond geometry (Å, °)

*D*—H⋯*A*	*D*—H	H⋯*A*	*D*⋯*A*	*D*—H⋯*A*
O2—H2⋯N2^i^	0.82	1.87	2.694 (2)	178
C2—H2*A*⋯O1^ii^	0.93	2.58	3.230 (2)	127
C8—H8⋯O2^iii^	0.93	2.48	3.392 (2)	165
C9—H9⋯O1^iv^	0.93	2.45	3.296 (2)	151

Symmetry codes: (i) 
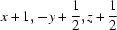
; (ii) 
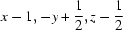
; (iii) 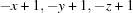; (iv) 
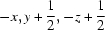
.

**Fig. 1.**
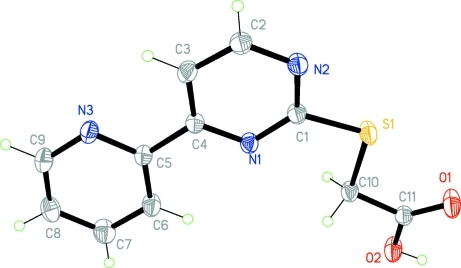
The structure of the title compound, showing 30% probability displacement ellipsoids.

**Fig. 2.**
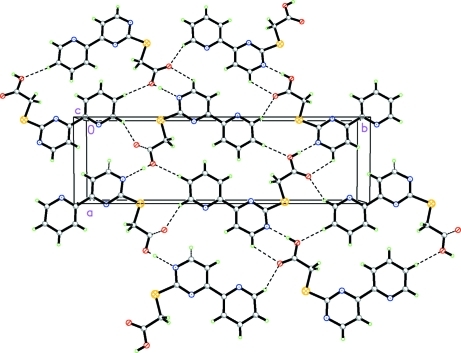
Perspective view of the crystal packing. Hydrogen bonding interactions are indicated with dashed lines.

## supplementary crystallographic information

### Comment

The rational dsign and assembly of new coordination polymers with with thioethers derived from 4-pyridinyl pyrimidine-2-thiol have received considerable attention in recent years (Dong *et al.*, 2009; Du *et al.*, 2004; Wang, 2011; Zhu *et al.*, 2009). Here we report the crystal structure of a newly synthesized compound derived from 4-(4-pyridinyl)pyrimidine-2-thiol.

The molecular structure of title compound is shown in Fig. 1 together with the atom-numbering scheme. The pyridine and pyrimidine rings are almost parallel with a dihedral angle of 6.7 (1)°. Molecules are linked by O-H···N hydrogen bonds into a chain running in direction [2 0 1]. C-H···O interactions join these chains into a two-dimensional network with base vectors [2 0 1] and [0 1 0] (equivalent to a sheet parallel to the (1 0 -2) lattice planes). Geometrical details are given in Table 1; a plot is given in Fig. 2.

### Experimental

All solvents and chemicals were of analytical grade and were used without further purification. The title compound was prepared by a similar procedure as reported in the literature (Dong *et al.*, 2009). To a solution of 4-(4-pyridinyl)pyrimidine-2-thiol (3.78 g, 20 mmol) and sodium hydroxide (0.80 g, 20 mmol) in water (30 ml), 2-bromoacetic acid (2.78 g, 20 mmol) in water (30 ml) was added. The mixture was stirred at room temperature for 4 h. Dilute hydrochloric acid was added to the reacted solution until the pH was about 4. Precipitates were filtered, washed by water and ethanol, and dried in vacuum. Single crystals suitable for X-ray diffraction were grown from a methanol solution by slow evaporation in air at room temperature.

### Refinement

All hydrogen atoms were geometrically positioned (C—H 0.93–0.97 Å) and refined as riding, with *U*_iso_(H)=1.2–1.5 *U*_eq_ of the parent atom.

### Crystal data

**Table:**

C_11_H_9_N_3_O_2_S	*F*(000) = 512.0
*M**_r_* = 247.28	*D*_x_ = 1.499 Mg m^−^^3^
Monoclinic, *P*2_1_/*c*	Mo *K*α radiation, λ = 0.71073 Å
Hall symbol: -P 2ybc	Cell parameters from 2524 reflections
*a* = 6.5722 (2) Å	θ = 1.8–27.5°
*b* = 22.4650 (8) Å	µ = 0.29 mm^−^^1^
*c* = 7.4314 (2) Å	*T* = 291 K
β = 93.237 (2)°	Block, pale yellow
*V* = 1095.45 (6) Å^3^	0.28 × 0.20 × 0.18 mm
*Z* = 4	

### Data collection

**Table:**

Bruker SMART CCD area-detector diffractometer	2524 independent reflections
Radiation source: fine-focus sealed tube	2116 reflections with *I* > 2σ(*I*)
graphite	*R*_int_ = 0.022
φ and ω scans	θ_max_ = 27.5°, θ_min_ = 1.8°
Absorption correction: multi-scan (*SADABS*; Bruker, 2000)	*h* = −8→8
*T*_min_ = 0.920, *T*_max_ = 0.950	*k* = −24→29
10868 measured reflections	*l* = −9→9

### Refinement

**Table:**

Refinement on *F*^2^	Primary atom site location: structure-invariant direct methods
Least-squares matrix: full	Secondary atom site location: difference Fourier map
*R*[*F*^2^ > 2σ(*F*^2^)] = 0.035	Hydrogen site location: inferred from neighbouring sites
*wR*(*F*^2^) = 0.096	H-atom parameters constrained
*S* = 1.05	*w* = 1/[σ^2^(*F*_o_^2^) + (0.0449*P*)^2^ + 0.289*P*] where *P* = (*F*_o_^2^ + 2*F*_c_^2^)/3
2524 reflections	(Δ/σ)_max_ = 0.001
155 parameters	Δρ_max_ = 0.21 e Å^−^^3^
0 restraints	Δρ_min_ = −0.24 e Å^−^^3^

### Special details

**Table:**

Experimental. The structure was solved by direct methods (Bruker, 2000) and successive difference Fourier syntheses.
Geometry. All e.s.d.'s (except the e.s.d. in the dihedral angle between two l.s. planes) are estimated using the full covariance matrix. The cell e.s.d.'s are taken into account individually in the estimation of e.s.d.'s in distances, angles and torsion angles; correlations between e.s.d.'s in cell parameters are only used when they are defined by crystal symmetry. An approximate (isotropic) treatment of cell e.s.d.'s is used for estimating e.s.d.'s involving l.s. planes.
Refinement. Refinement of *F*^2^ against ALL reflections. The weighted *R*-factor *wR* and goodness of fit *S* are based on *F*^2^, conventional *R*-factors *R* are based on *F*, with *F* set to zero for negative *F*^2^. The threshold expression of *F*^2^ > σ(*F*^2^) is used only for calculating *R*-factors(gt) *etc*. and is not relevant to the choice of reflections for refinement. *R*-factors based on *F*^2^ are statistically about twice as large as those based on *F*, and *R*- factors based on ALL data will be even larger.

### Fractional atomic coordinates and isotropic or equivalent isotropic displacement parameters (Å^2^)

**Table:**

	*x*	*y*	*z*	*U*_iso_*/*U*_eq_	
C1	−0.0901 (2)	0.34978 (6)	0.26132 (19)	0.0342 (3)	
C2	−0.3668 (2)	0.39680 (7)	0.1300 (2)	0.0412 (4)	
H2A	−0.4973	0.3959	0.0747	0.049*	
C3	−0.2718 (2)	0.45062 (7)	0.1562 (2)	0.0391 (3)	
H3	−0.3370	0.4860	0.1229	0.047*	
C4	−0.0745 (2)	0.45042 (6)	0.23433 (19)	0.0327 (3)	
C5	0.0431 (2)	0.50633 (6)	0.25998 (19)	0.0333 (3)	
C6	0.2462 (2)	0.50591 (7)	0.3208 (2)	0.0427 (4)	
H6	0.3120	0.4702	0.3493	0.051*	
C7	0.3493 (3)	0.55934 (8)	0.3385 (2)	0.0511 (4)	
H7	0.4862	0.5602	0.3778	0.061*	
C8	0.2464 (3)	0.61115 (7)	0.2972 (2)	0.0493 (4)	
H8	0.3113	0.6478	0.3096	0.059*	
C9	0.0444 (3)	0.60757 (7)	0.2367 (3)	0.0490 (4)	
H9	−0.0242	0.6428	0.2078	0.059*	
C10	0.2527 (2)	0.30146 (7)	0.4321 (2)	0.0380 (3)	
H10A	0.3269	0.3242	0.3463	0.046*	
H10B	0.2369	0.3259	0.5380	0.046*	
C11	0.3674 (2)	0.24555 (7)	0.4832 (2)	0.0365 (3)	
N1	0.01710 (17)	0.39939 (5)	0.28821 (16)	0.0340 (3)	
N2	−0.27844 (18)	0.34541 (6)	0.18112 (18)	0.0391 (3)	
N3	−0.0580 (2)	0.55666 (6)	0.21748 (19)	0.0432 (3)	
O1	0.30963 (19)	0.19622 (5)	0.4458 (2)	0.0623 (4)	
O2	0.53762 (17)	0.25707 (5)	0.57657 (19)	0.0544 (3)	
H2	0.5945	0.2257	0.6057	0.082*	
S1	0.00702 (6)	0.280700 (17)	0.33349 (6)	0.04496 (15)	

### Atomic displacement parameters (Å^2^)

**Table:**

	*U*^11^	*U*^22^	*U*^33^	*U*^12^	*U*^13^	*U*^23^
C1	0.0331 (7)	0.0315 (7)	0.0371 (8)	−0.0008 (6)	−0.0044 (6)	0.0020 (6)
C2	0.0329 (7)	0.0395 (8)	0.0500 (9)	0.0000 (6)	−0.0100 (6)	0.0044 (7)
C3	0.0357 (7)	0.0329 (8)	0.0477 (9)	0.0046 (6)	−0.0053 (6)	0.0035 (6)
C4	0.0344 (7)	0.0297 (7)	0.0338 (7)	0.0004 (5)	0.0004 (6)	0.0001 (5)
C5	0.0373 (7)	0.0279 (7)	0.0345 (7)	0.0011 (6)	−0.0004 (6)	−0.0005 (6)
C6	0.0425 (8)	0.0330 (8)	0.0512 (9)	−0.0010 (6)	−0.0106 (7)	0.0027 (7)
C7	0.0470 (9)	0.0454 (10)	0.0591 (11)	−0.0091 (7)	−0.0137 (8)	−0.0012 (8)
C8	0.0612 (10)	0.0321 (8)	0.0543 (10)	−0.0104 (7)	0.0000 (8)	−0.0057 (7)
C9	0.0564 (10)	0.0281 (8)	0.0628 (11)	0.0042 (7)	0.0045 (8)	−0.0008 (7)
C10	0.0340 (7)	0.0297 (8)	0.0491 (9)	0.0002 (6)	−0.0078 (6)	0.0004 (6)
C11	0.0324 (7)	0.0313 (8)	0.0452 (8)	0.0003 (6)	−0.0041 (6)	−0.0005 (6)
N1	0.0328 (6)	0.0284 (6)	0.0399 (7)	−0.0004 (5)	−0.0052 (5)	0.0024 (5)
N2	0.0347 (6)	0.0331 (7)	0.0482 (7)	−0.0028 (5)	−0.0096 (5)	0.0040 (5)
N3	0.0418 (7)	0.0286 (7)	0.0587 (8)	0.0041 (5)	0.0000 (6)	0.0007 (6)
O1	0.0526 (7)	0.0291 (6)	0.1011 (11)	−0.0001 (5)	−0.0329 (7)	−0.0024 (6)
O2	0.0424 (6)	0.0322 (6)	0.0852 (9)	0.0028 (5)	−0.0269 (6)	−0.0038 (6)
S1	0.0384 (2)	0.0272 (2)	0.0670 (3)	−0.00354 (15)	−0.01691 (18)	0.00762 (17)

### Geometric parameters (Å, °)

**Table:**

C1—N1	1.3276 (18)	C7—C8	1.372 (2)
C1—N2	1.3466 (18)	C7—H7	0.9300
C1—S1	1.7507 (15)	C8—C9	1.380 (2)
C2—N2	1.3377 (19)	C8—H8	0.9300
C2—C3	1.369 (2)	C9—N3	1.331 (2)
C2—H2A	0.9300	C9—H9	0.9300
C3—C4	1.3909 (19)	C10—C11	1.503 (2)
C3—H3	0.9300	C10—S1	1.7964 (14)
C4—N1	1.3453 (17)	C10—H10A	0.9700
C4—C5	1.4815 (19)	C10—H10B	0.9700
C5—N3	1.3401 (18)	C11—O1	1.1991 (18)
C5—C6	1.385 (2)	C11—O2	1.3085 (17)
C6—C7	1.381 (2)	O2—H2	0.8200
C6—H6	0.9300		
			
N1—C1—N2	126.48 (13)	C7—C8—C9	118.45 (15)
N1—C1—S1	121.11 (10)	C7—C8—H8	120.8
N2—C1—S1	112.41 (10)	C9—C8—H8	120.8
N2—C2—C3	122.31 (13)	N3—C9—C8	123.84 (15)
N2—C2—H2A	118.8	N3—C9—H9	118.1
C3—C2—H2A	118.8	C8—C9—H9	118.1
C2—C3—C4	117.60 (13)	C11—C10—S1	108.22 (10)
C2—C3—H3	121.2	C11—C10—H10A	110.1
C4—C3—H3	121.2	S1—C10—H10A	110.1
N1—C4—C3	121.20 (13)	C11—C10—H10B	110.1
N1—C4—C5	117.57 (12)	S1—C10—H10B	110.1
C3—C4—C5	121.23 (13)	H10A—C10—H10B	108.4
N3—C5—C6	122.60 (14)	O1—C11—O2	123.76 (14)
N3—C5—C4	115.89 (12)	O1—C11—C10	124.47 (13)
C6—C5—C4	121.49 (13)	O2—C11—C10	111.76 (12)
C7—C6—C5	118.94 (15)	C1—N1—C4	116.48 (12)
C7—C6—H6	120.5	C2—N2—C1	115.86 (12)
C5—C6—H6	120.5	C9—N3—C5	117.29 (14)
C8—C7—C6	118.87 (15)	C11—O2—H2	109.5
C8—C7—H7	120.6	C1—S1—C10	101.46 (7)
C6—C7—H7	120.6		

### Hydrogen-bond geometry (Å, °)

**Table:**

*D*—H···*A*	*D*—H	H···*A*	*D*···*A*	*D*—H···*A*
O2—H2···N2^i^	0.82	1.87	2.694 (2)	178
C2—H2A···O1^ii^	0.93	2.58	3.230 (2)	127
C8—H8···O2^iii^	0.93	2.48	3.392 (2)	165
C9—H9···O1^iv^	0.93	2.45	3.296 (2)	151

Symmetry codes: (i) *x*+1, −*y*+1/2, *z*+1/2; (ii) *x*−1, −*y*+1/2, *z*−1/2; (iii) −*x*+1, −*y*+1, −*z*+1; (iv) −*x*, *y*+1/2, −*z*+1/2.
